# Evaluation of an ultrasound bladder scanner in supine and standing position

**DOI:** 10.1002/acm2.13424

**Published:** 2021-10-22

**Authors:** Frederik Crop, Pauline Comte, Florence Le Tinier, David Pasquier, Xavier Mirabel

**Affiliations:** ^1^ Department of Medical Physics Centre Oscar Lambret Lille France; ^2^ Academic Department of Radiotherapy Centre Oscar Lambret Lille France

**Keywords:** bladder filling, BladderScan, BVI 9400, radiotherapy

## Abstract

**Purpose:**

This study examined the performance of a bladder volume measuring device, the BladderScan (BS) BVI9400. The use of the BS offers the possibility of assessing the bladder volume before positioning the patient and performing the daily image‐guided radiotherapy procedure. Patients often cannot lie down before entering the treatment vault. Therefore, the BS was also assessed in a standing position.

**Methods:**

The repeatability precision was first evaluated, which is the variability of immediate repeated measures of the BS with same operator and subject. This was followed by the reproducibility precision of the BS in which the operator and subjects differ. Finally, the trueness was evaluated in terms of fixed and proportional bias of the results by applying weighted least‐squares fitting. Note that 53 and 85 patient measurements were carried out in supine and standing position, respectively, each consisting of three repeated BS measurements. These were compared with the computed tomography (CT)‐delineated bladder volume.

**Results:**

Repeatability was dependent on measurement value (heteroscedasticity) with *σ*_repeatability_ (BS) = ±15 cm^3^ ± 10%. However, the total agreement between BS and CT was low with the 95% limits of agreement (LOAs) exceeding ±200 cm^3^ due to poor patient reproducibility and presence of fixed and proportional bias. Only in the best case of male patients in the supine position, three BS measurements, and correction for the fixed and proportional bias, 95% LOAs of [–147, +114] cm^3^ were obtained between CT and BS.

**Conclusion:**

The agreement of the BVI9400 BS with CT was found to be too low for radiotherapy applications.

## 1 INTRODUCTION

Bladder volume affects the clinical target volume (CTV) position and the resulting dose coverage of the CTV for individual patients.^1^ If the bladder volume and/or shape during the treatment fraction is not equivalent to the volume on planning computed tomography (CT), the target volume can be underdosed or the bladder overdosed. Several approaches can be used to manage the bladder volume. The first is daily adaptation, as applied in MR‐linac^2^ or Cone‐Beam CT (CBCT)‐ or Mega Voltage CT (MVCT)‐based online adaptation approach.^3^ However, this approach is not at all widespread. Another approach is to use a “plan of the day”: multiple treatment plans for empty, intermediate, and full bladder volumes are created before treatment.^4^, ^5^ Each fraction, the radiation therapist (RTT) then selects the best‐suited radiation therapy plan.^6^ However, the choice of the best‐suited plan is subject to a large interrater variability.^5^ This approach is not commonly used as it requires additional planning CTs with different bladder filling and subsequent contouring and treatment plans. Another approach is to manage the bladder volume as effectively as possible by following strict protocols. However, patients do not always adhere100% to the protocol, the bladder volume will be different throughout a radiotherapy course,^7^ or the schema will not be maintained if the time between preparation and treatment is not respected. The use of the bladder‐filling measuring device before starting CBCT/MVCT or even before positioning the patient offers a possible solution: if the bladder volume is comparable to the initial bladder volume for the planning CT, the bladder volume itself should not pose an issue. If the volume is not within tolerances, the patient should void part of the bladder or wait and another patient can be treated meanwhile.

Eminowicz et al.^1^ reported that the bladder volume during treatment should ideally be in an interval of [–50; +150] cm^3^ compared to original planning CT volume. Therefore, the BladderScan (BS) should achieve higher agreement.

Several groups reported correlations for different BS devices: BVI 3000,^8^, ^9^ BVI6100,^10^ and bladdermanager.^11^ Brouwer et al.^12^ assessed the precision of the BVI 9400 (Verathon Inc, Bothell, WA, USA) at the start of our study. They found an overestimation of bladder volume by 17.5% when compared to catheterization. However, a fixed bias can be corrected for with a new calibration.

The device was also assessed with the patient in a standing position as the radiotherapy workflow can benefit from a bladder scan with the patient standing in the undressing room. Often, there is no place for a table for the patient to lie down before entering the radiotherapy treatment room. The bladder shape will of course be different when standing, but the volume remains constant. This strategy could eliminate multiple positioning and CT scans of patients with bladder volumes that are too large or too small.

In this study, the technical performance of the BVI 9400 compared to the contoured bladder volume of the CT immediately after the bladder scan was evaluated in detail with the patient both standing and in supine position. Finally, all uncertainty and bias evaluations are performed following Quantitative Imaging Biomarker Association (QIBA) guidelines^13^, ^14^, ^15^ in order to obtain the correct uncertainty intervals when measuring a bladder volume with the BVI 9400.

## 2 MATERIALS AND METHODS

### 2.1 BladderScan BVI 9400

**FIGURE 1:**
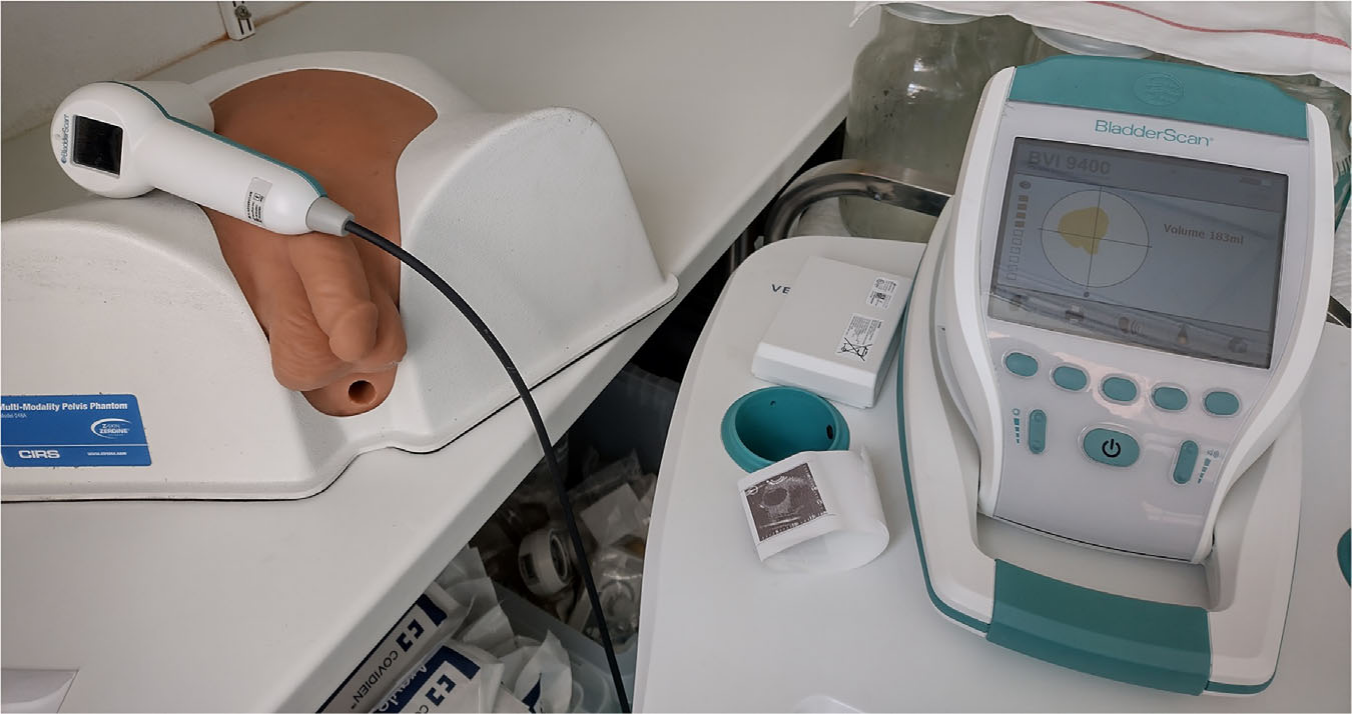
BladderScan BVI 9400 and CIRS multimodality 048A phantom. The screen displays if the bladder is correctly centered

A BVI 9400 (Figure 1) ultrasound scan is taken by positioning the probe, with application of ultrasound gel, approximately 3 cm above the symphysis. The probe should be aimed toward the bladder, but there is also a visual simplified graphical aid in order to verify bladder “centering.” The BVI 9400 uses neural network harmonics that applies multispectral three‐dimensional analysis. This should not only accelerate but also improve the detection of the bladder and discern better between the uterus and the bladder. The BS has three modes: (a) male or female with hysterectomy, (b) female without hysterectomy, and (c) small child.

The manufacturer indicates an “accuracy”^1^ of ±15% ± 15 cm^3^ on the manufacturer's phantom and bladder volume range of 0–999 cm^3^. When taking this as a 95% confidence interval (CI), a measure of 200 cm^3^ should correspond to a real value between 155 and 245 cm^3^.

### 2.2 CT scans and contouring

As a CT scan was required for dosimetry planning for radiotherapy, no additional CT scans were required. The CT (Toshiba) scan characteristics were as follows: slice thickness 3 mm, resolution 512 × 512 with pixel size between 0.65 and 1 mm, and kVp 120 keV. The TomoTherapy (Accuray Inc, Sunnyvale, CA, USA) MVCT characteristics were slice thickness 3 mm and 512 × 512 resolution with pixel size 0.76 mm using the photon beam degraded to 3 MV. The scan volume encompassed the whole pelvis. Manual detailed contouring of the bladder was performed by radiotherapy oncologists using Raystation (Raysearch, Stockholm, Sweden) using ESTRO reference guidelines. A 3 mm isotropic size reduction was applied in order to subtract the bladder wall.^16^

### 2.3 Patients

**FIGURE 2:**
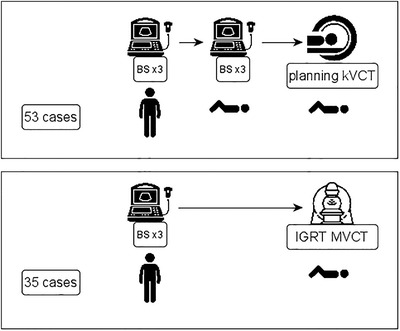
Summary of measurements performed. Icons from the Noun project (see Acknowledgments)

The study setup is depicted in Figure 2. There were 53 sets of three measurements in both standing and supine position, followed by kilo Voltage Computed Tomography. There were also 35 additional sets of three measurements in standing position followed by MVCT. They concerned 53 patients of which 22 were women without hysterectomy. No specific selection criteria were applied. Three sets of three successive BS measurements with the patient standing up resulted in null BS measurements and were removed. The subject of this study was the correlation between BS measurement and CT contoured bladder volume, thus no interfraction correlation was sought. The bladder preparation protocol aims to obtain a comfortable bladder filling between 100 and 150 cm^3^: the patients are instructed to void the bladder 1 h before the examination or treatment fraction and drink 200 cm^3^ of water.

### 2.4 Agreement and nomenclature

The QIBA workgroup methodology foranalysis^13^, ^14^, ^15^ was applied, following ISO 5725–1 for nomenclature.^17^ Agreement was defined in terms of precision and trueness:

1. Precision (random components)
  - Repeatability: repeated measures, same operator and subject: *σ*_repeat_.
  - Reproducibility: different users and subject: *σ*_reprod_: *σ* ^2^_reprod_ = *σ* ^2^_repeat_ + *σ* ^2^_operator+subject_.
2. Trueness
  - Fixed bias or common systematic error.
  - Proportional bias.

We refer to the Supporting Information and ISO for exact definitions. Precision can be divided in repeatability and reproducibility. Even though close to the “true value,” the CT‐based bladder volume is not a real true value, and as of such, the term “agreement” is used instead of “accuracy.”

Trueness is defined as the difference between the mean measured value and the measurand, which can consist of a true value or a reference value. The bias can be (a) fixed or constant, (b) proportional, or (c) nonfixed/nonlinear, of which an example is given by Sullivan et al.^14^

### 2.5 Precision: Repeatability: Within‐subject standard deviation *σ*_repeat_ and heteroscedasticity

The repeatability was first assessed on a CIRS 048A multimodality male phantom (Figure 1) by performing 20 measurements for general and female mode.

The patient repeatability conditions were as follows: same operator, same patient with same bladder filling, same apparatus, and three immediate measurements. The measurement results were verifiedfor heteroscedasticity using a Breusch–Pagan test from the lm package on a *p* < 0.05 (The r project^18^). Heteroscedasticity corresponds to a different standard deviation of the results, depending on the value of the measured value. The results were binned in order to improve robustness of the fit.

### 2.6 Precision: Reproducibility

#### 2.6.1 Precision: Reproducibility (operator): CT‐based bladder volume *σ*_reprod, operator_ (CT)

We refer to the study of Meijer et al.^19^ for bladder contouring interoperator variability on CT: they showed a *σ*_reprod, operator_ (CT) of 11.8 cm^3^ over a range of 50–250 cm^3^. Confirmation of their findings was sought by analyzing results of a previous study^20^: for this study, 14 operators contoured two pelvic treatment patients. The operators were radiation oncologists of different centers, thus representing different training, experience, and habits. The geometrical accuracy of the CT scanner was under standard radiotherapy quality control, requiring <1 mm accuracy.

#### 2.6.2 Precision: Reproducibility (operator): BS‐based bladder volume *σ*_reprod, operator_ (BS)

The interoperator reproducibility *σ*_reprod, operator_ (BS) of the BS was verified by comparing the measurements of the three main users representing >50% of all patient measurements. Each performed 10 successive measurements of two subjects. Systematic differences (*p* < 0.05) in the mean were checked by a two‐sided Student's test, but also in the median by a Brown‐Forsythe, Levene‐type, test and finally differences in variability by an *F* test and an ANOVA test. One outlier BS measurement was removed: 200 cm^3^ for a mean measurement of 393 cm^3^.

#### 2.6.3 Precision: Reproducibility (operator and subject): BS‐based bladder volume *σ*_reprod_ (BS)

Bland–Altman plots were used to assess the reproducibility of the BS following the methodology described previously^21^, ^22^, ^23^, ^24^ by evaluating the limits of agreement (LOAs). The *σ*_reprod_ (CT) and uncertainty in the trueness of the CT‐based volume are indirectly taken into account using the Bland–Altman plots as the mean of both BS and CT measures on the *X*‐axis.

### 2.7 Trueness: Fixed and proportional bias

The trueness of the CT contoured volume and BS measurement was verified first on the CIRS multimodal 048A phantom (Figure 1), for which CIRS provided a calibration certificate indicating a 177 cm^3^ bladder volume in the phantom. For patients, a true value does not exist and the CT volume is considered the reference value close to the true value. This is also valid as the CT volume is used throughout the radiotherapy planning and subsequent IGRT treatments as reference. As both *X* (CT) and *Y* (BS) values express uncertainty, a model IIA linear regression such as weighted least products should be required. However, in case the reproducibility of CT‐based bladder volume *σ*_reprod_ (CT) is two to three times lower than the reproducibility of the BS, a weighted linear regression can be applied using the previously described heteroscedasticity to quantify a fixed and proportional bias.^25^, ^26^

In the ideal case, there should be a perfect linear relationship with intercept 0 (no fixed bias) and slope 1 (no proportional bias: perfect linearity) if both the BS apparatus and the CT agree. As the manufacturer's intended use of the BS was in the supine position, both an additional fixed and proportional bias can occur for standing patients: the shape of the bladder will be different when standing. However, the bladder volume should stay the same and correspond to the volume in supine position.

When intending to use the BS as a measure of the CT bladder volume, one needs to consider correctly the uncertainty intervals: the relationship must be inverted, which corresponds to the well‐known “calibration problem.” The uncertainty interval for this inverse prediction was calculated with the inverse.predict function from the chemCal package from the R project.^18^ Thus, the inverse prediction uncertaintyinterval does not refer to *σ*_reprod_ (BS), but the practical use of the BS as a predictor of CT bladder volume. An example of this reading is given in Figure S1. A new calibration was conductedfor both supine and standing position in order to improve the agreement as Brouwer et al.^12^ mentioned that a systematic bias can occur in supine position.

## 3 RESULTS

### 3.1 Precision: Repeatability: Within‐subject standard deviation *σ*_repeat_ (BS) and heteroscedasticity

**FIGURE 3:**
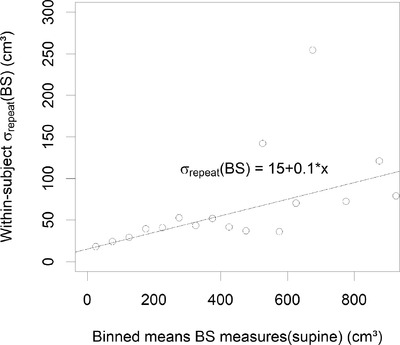
Heteroscedasticity of BladderScan measurements: Dependence of within‐subject variability *σ*_repeat_ (BS) on measurement results

The (male) phantom tests resulted in a *σ*_repeat_ (BS, phantom) = 24 and 13 cm^3^ for the general and female mode, respectively. The Breusch–Pagan test on patient data resulted in significant *p* values for heteroscedasticity of the data when the mean of the BS measurement values was used as a reference value. These results are shown in Figure 3 where binned results were used in order to improve the robustness of fit. This resulted in the linear function of the *σ*_repeat_ (BS) = 15 cm^3^ + 10% (measurement). This binned *σ*_repeat_ (BS) value was not significantly different between male and female subjects or standing or supine position.

### 3.2 Precision: Reproducibility

#### 3.2.1 Precision: Reproducibility (operator): CT‐based bladder volume

The results of Meijer et al.,^19^ *σ*_reprod, operator_ (CT) = 11.8 cm^3^, were verified on contouring data of 14 radiation oncologists and this resulted in a *σ*_reprod, operator_ (CT) = 7.3 and 9.1 cm^3^ for two patients with mean bladder volume of 151 and 74 cm^3^, respectively.

#### 3.2.2 Precision: Reproducibility (operator): BS‐based bladder volume *σ*_reprod, operator_ (BS)

There was no statistically significant difference (*p* > 0.05) in the mean, the median, or the variability of each of the main three operators. The operator dependent reproducibility fora bladder volume of 393 and 232 cm^3^ was *σ*_reprod, operator_ (BS) 36 and 28 cm^3^, respectively. The operator dependent reproducibility uncertainty for the BS was thus about three times larger than the CT based uncertainty.

#### 3.2.3 Precision: Reproducibility (operator and patient): Bladder scan

**FIGURE 4:**
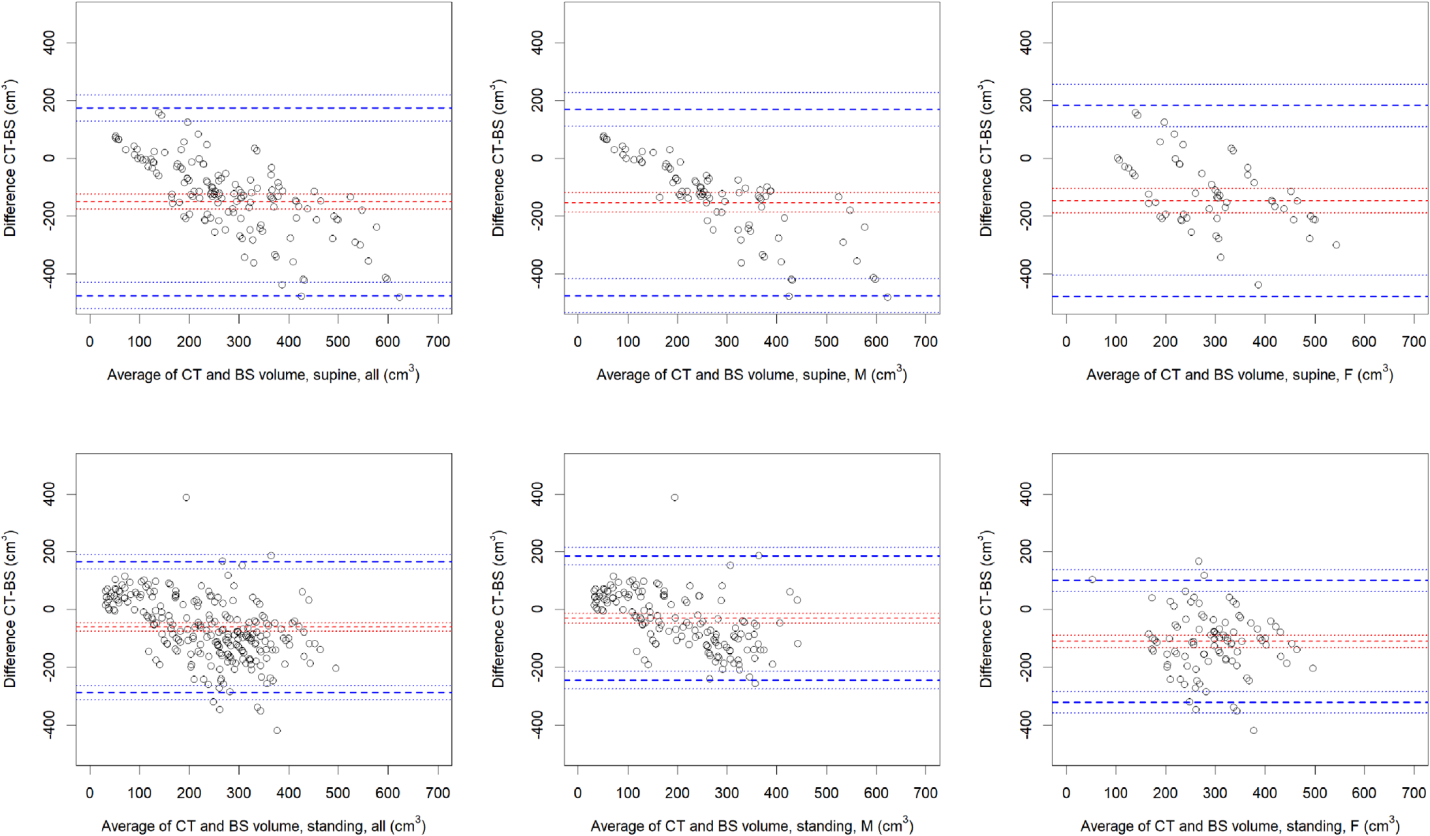
Bland–Altman plots of all results: Internal bladder volume on CT without bladder wall (3 mm subtraction) versus BVI9400

The Bland–Altman plots of the bladder wall‐subtracted CT volume versus BS volume are shown in Figure 4. The 95% LOAs, depicted as the blue dashed lines, are substantial in all cases in the order of ±200 cm^3^. The dashed red lines represent the fixed bias, which is substantial in most cases. A proportional bias can be observed in supine position: the values are distributed differently around the fixed bias with increasing *X*‐axis values. These fixed and proportional biases are quantified and corrected for in the next paragraph.

#### 3.2.4 Trueness: Fixed and proportional bias

The bladder CT contoured volume of the CIRS phantom was 180 cm^3^, close to the CIRS‐calibrated 177 cm^3^. The obtained mean BS values were 195 and 198 cm^3^ for general and female mode, respectively.

**FIGURE 5:**
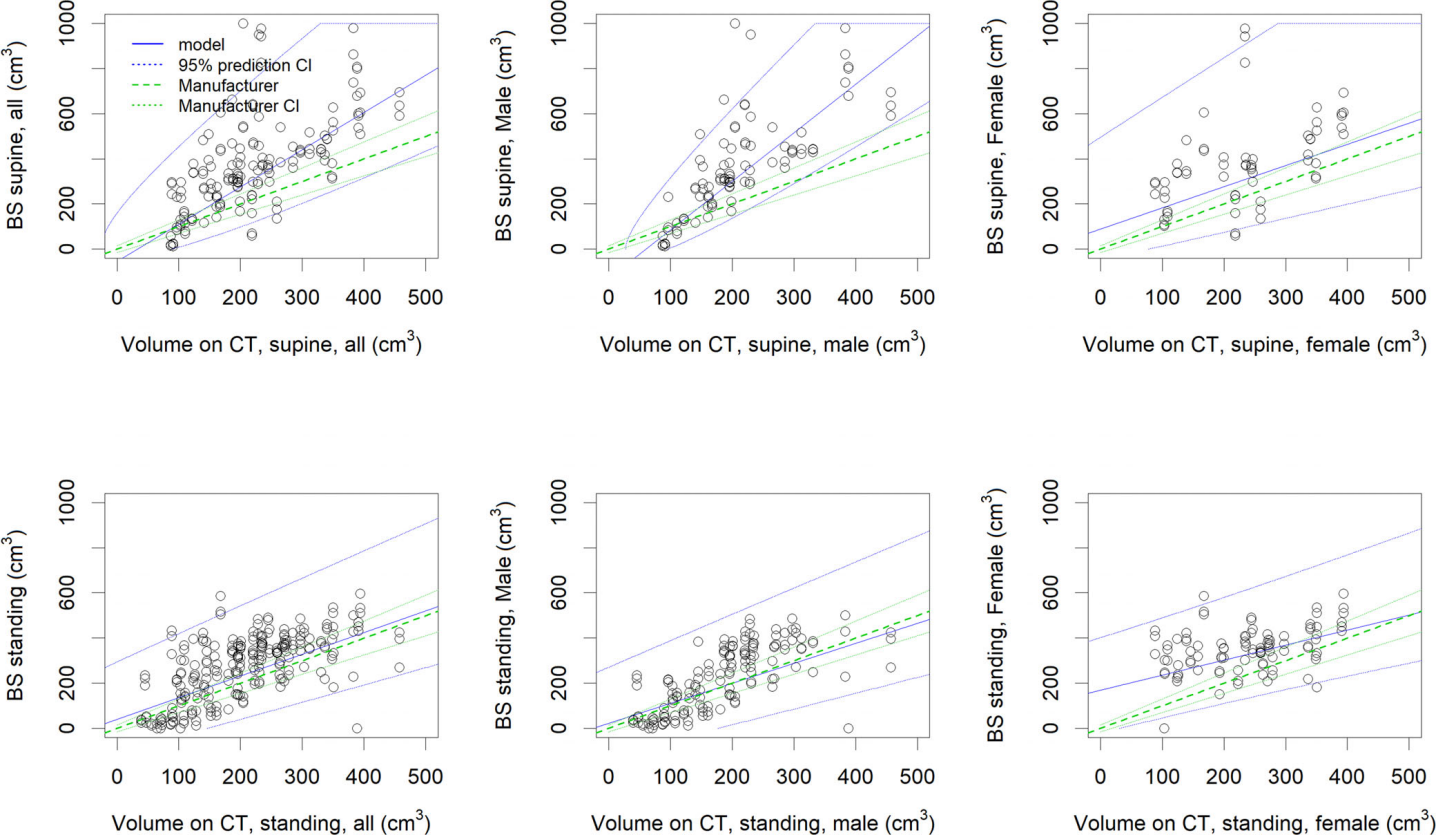
Linearity and bias for BladderScan (BS) for a single measurement: Internal bladder volume without bladder wall. The blue uncertainty intervals correspond to the inverse prediction 95% confidence intervals (CI): when measuring a BS value (value on the *Y*‐axis), the actual predicted value will correspond to the indicated interval on the *X*‐axis (see also Figure S1). The green lines correspond to the constructor's stated uncertainty (CI, confidence interval)

The results of the inverse prediction correcting for fixed and proportional bias are depicted in Table 1 and Figure 5. These show that both fixed bias (intercept ≠ 0) and proportional bias (slope ≠ 1) are present in most cases, except for male standing patients. The fitted blue curves correspond to the inverse prediction process: when measuring a BS value, the corresponding predicted CT value is then obtained on the *X*‐axis as depicted in Figure S1. The 95% confidence interval follows the same logic, using the dotted blue lines. The correlation in Table 1 between the CT values and BS measured values is between 0.22 and 0.75 for adjusted *R* ^2^, and the Spearman correlation coefficient is between 0.38 and 0.82.

**TABLE 1:** Fixed and proportional bias results for BS versus CT internal bladder volume: Bladder—3‐mm bladder wall

	Supine			Standing		
	All	M	F	All	M	F
Fixed bias (intercept)	–53	–130	90	23.7	–18	260
Standard error	23	22.6	48	14.5	15	26
Conf int (0.95)	[–99; –7]	[–175; –85]	[–6; 186]	[–5; 52]	[–47; 12]	[209, 312]
Proportional bias (slope)	1.65	2.15	0.94	1.13	1.23	0.35
Standard error	0.12	0.13	0.21	0.07	0.08	0.1
Conf int (0.95)	[1.41; 1.88]	[1.90; 2.40]	[0.51; 1.36]	[0.99; 1.27]	[1.06; 1.39]	[0.14; 0.56]
Adjusted *R* ^2^ of wls fit	0.55	0.75	0.23	0.37	0.35	0.22
Spearman correlation coeff	0.71	0.82	0.58	0.63	0.71	0.38

Abbreviations: F, Female; M, Male; wls, weighted least squares.

## 4 DISCUSSION

### 4.1 Precision: Repeatability: Within‐subject standard deviation and heteroscedasticity

The repeatability on phantom showed a significant difference between female and male mode. However, the phantom was a “male” phantom and as of such no “virtual uterus” was present and could possibly perturb the algorithm.

The patient‐related *σ*_repeat, subject_ (BS) found was comparable to the overall “accuracy” provided by the manufacturer. This value could be reduced by taking the mean of three successive measurements.

### 4.2 Precision: Reproducibility BS (subject and user)

In the Bland–Altman plots in Figure 4, the 95% LOAs were higher than ±200 cm^3^. The origin can most likely be found in an issue with the trueness and *σ*_reprod, subject_ (BS) for patients as the *σ*_reprod, operator_ (BS) was found to be constant between the main three operators.

### 4.3 Trueness: Bias and inverse prediction

The trueness on phantom showed the same results as indicated by Brouwer et al.^12^: a (fixed) bias of +18 cm^3^ was present on phantom, indicating a new calibration is thus required. A proportional bias can only be evaluated for patients as there are no phantoms with the full range available.

The patient results in Figure 5 show the uncertainty intervals of the predicted CT‐based volumes, based on a BS measurement. The blue lines correspond to the inverse prediction uncertainty interval. When obtaining a BS measurement, it is taken on the *Y*‐axis and the corresponding “reference” CT value and 95% confidence interval can be read on the *X*‐axis following the blue lines indicated in the graphs. The results show that slope = 1 is not respected; thus, a proportional bias correction should be applied to the BVI 9400 measure.

Less proportional bias, slope = 1, was found for patients standing up than those in the supine position. However, supralinearity in supine position results in better predictive confidence intervals in practice when corrected for nonproportional bias.

Only in the best case of male patients in supine position, taking three measurements and correcting for the bias with the BVI 9400, a 95% CI of [116, 289] cm^3^ was obtained for a BS measurement of 200 cm^3^. When considering the interval range of 100–500 cm^3^, LOAs of [–147, 114] cm^3^ were obtained, which is not compatible with the required [–50; 150] cm^3^ interval.^1^

A possible bias in our analysis could be bladder filling between the BS measurement and CT acquisition: a better proportional bias but a higher fixed bias (results not shown here) when considering the total bladder volume was achieved, as did Ahmad et al.^27^ However, this cannot explain the results, as one expects the inverse: the BS measurement was before the CT. Thus, the CT volume should be larger than the BS value.

A limitation of this study is due to the different contrast mechanisms in both imaging modalities. The BS is based on differences in acoustic impedance between urine and the bladder wall. The CT contoured volume is based on the bladder including bladder wall, of which a uniform margin of 3 mm is subtracted. However, the bladder wall thickness canvary and exhibits a 1 mm standard deviation and has a small correlation with volume of –0.001 mm/cm^3^.^16^ The former would be expressed as a variability in the CT‐based results and the latter as a small nonconstant bias.

Our study investigated only the volume of the bladder and not the shape, which has an important impact on the radiotherapy plan. The main factor in bladder shape, however, is the bladder filling.^28^, ^29^ There is also an interaction with rectal filling, leading to a shift/rotation of the bladder but not an important different bladder shape.^28^ The small bowel filling can also lead to a difference in shape in the cranial part of the bladder.^29^ Finally, there is also a variability in shape for premenopausal patients due to uterus size differences with the female cycle.^29^ The possibility of verifying the bladder volume would improve the main factor, which is the filling.

Even though other authors have achieved better results with previous versions of the BVI, our results show an issue with the BVI 9400. Brouwer et al.^12^ identified a bias with the BVI 9400 type, which was quantified more in detail and corrected for in this work. The uncertainty stated by the manufacturer corresponds to *σ*_repeat_ (BS) and not the total accuracy/agreement. A possible cause would be an error or instability in the underlying algorithm, which is a neural network/harmonic model trained on simulated body fluids and body tissues, to calculate the three‐dimensional echographic volume.

Finally, the BS BVI 9400 could still possibly be used to decide on the “plan of the day”: although not very accurate, this could imply the same probability as the RTT selecting the correct plan of the day.

## 5 CONCLUSION

Although older versions of the BladderScan BVI have been reported to perform better, our results indicate that the BVI 9400 should not be used as a device to assess bladder volume for radiotherapy. Even with fixed and proportional bias correction, the agreement is too low to determine the correct bladder filling.

## CONFLICT OF INTEREST

The authors declare no conflict of interest.

## AUTHOR CONTRIBUTIONS

All authors designed the study and reviewed the manuscript. Frederik Crop performed statistics analysis. Frederik Crop and Pauline Comte performed technical testing. Florence Le Tinier, David Pasquier, and Xavier Mirabel assisted in the clinical implementation.

## Supporting information

Supporting Information

## Data Availability

Research data are not publicly available but are available from the corresponding author on reasonable request.

## Footnotes

1 Verathon. BVI 9400 User's Manual. The manufacturer did not respond to a query regarding the details of the stated accuracy/agreement concerning the uncertainty intervals and trueness or precision. We expect this to be *k* = 2 for total accuracy/agreement and a 95% confidence interval (CI).
