# Supplementary material for: Evaluation of an ultrasound bladder scanner in supine and standing position
Source: J Appl Clin Med Phys. 2021 Oct 22;22(12):194–202. doi: 10.1002/acm2.13424 (PMC8664133; doi:10.1002/acm2.13424)
Supplement: Supplementary file 1 — Supporting Information [file ACM2-22-194-s001.zip › acm213424-sup-0001-SuppMat.docx]

**Supplementary file : Definitions**

**Agreement**(definition QIBA): the degree of closeness between measurements made on the same experimental unit.

Definitions from the International vocabulary of basic and general terms in metrology (VIM) :

B.2.3 **True value (of a quantity)** : value consistent with the definition of a given particular quantity

2.13 **Measurement accuracy** : closeness of agreement between a measured quantity value and a true quantity value of a measurand – is not a quantity

2.14 **Measurement Trueness** : closeness of agreement between the average of an infinite number of replicate measured quantitiy values and a reference quantity value – is related to systematic measurement error

2.15 **Measurement precision** : closeness of agreement between indications or measured quantity values obtained by replicate measurements on the same or similar objects under specified conditions – used to define repeatability, intermediate measurement precision and measurement reproductibility

Note 4 (from ISO): Sometimes « measurement precision » is erroneously used to mean measurement accuracy
