# Supplementary figures and images for: Evaluation of an ultrasound bladder scanner in supine and standing position
Source: J Appl Clin Med Phys. 2021 Oct 22;22(12):194–202. doi: 10.1002/acm2.13424 (PMC8664133; doi:10.1002/acm2.13424)

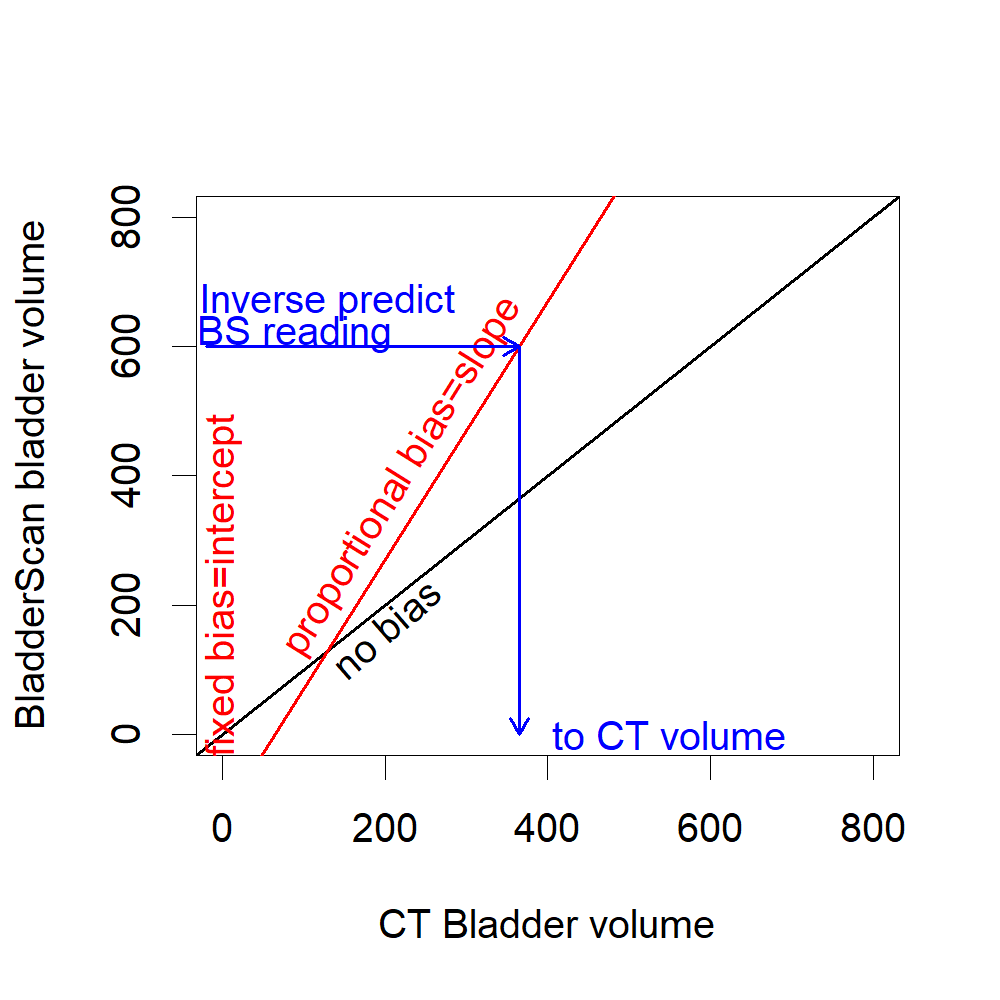

Supplement: Supplementary file 1 — Supporting Information [file ACM2-22-194-s001.zip › Supp_fig.png]
